# Addressing the challenge of antimicrobial resistance

**DOI:** 10.1038/s43856-025-00758-1

**Published:** 2025-03-06

**Authors:** Timothy M. Rawson, Luke SP Moore, Mohammed Lamorde

**Affiliations:** 1 Centres for Antimicrobial Optimisation Network, Imperial College London, London, UK; 2 Health Protection Research Unit in Healthcare Associated Infections and Antimicrobial Resistance, Imperial College London, London, UK; 3 The David Price Evans Global Health & Infectious Diseases Group, The University of Liverpool, Liverpool, UK; 4 Clinical Infection Department, Chelsea and Westminster Hospital NHS Foundation Trust, London, UK; 5 Infectious Diseases Institute, College of Health Sciences, Makerere University, Kampala, Uganda

**Keywords:** Antimicrobial resistance

## Abstract

Antimicrobial resistance (AMR) is a global health challenge that requires cross-disciplinary collaboration to mitigate its impact on human health. We discuss some of the topical advances in the field, highlighting the AMR collection, which brings attention to the problem of AMR and suboptimal antimicrobial use in human medicine.

Antimicrobial resistance (AMR) occurs when organisms that were previously susceptible to an antimicrobial become resistant to its killing effects. AMR is a global health emergency that was associated with nearly 5 million deaths in 2019^1^. It is estimated that by 2050 without further intervention AMR will cause more deaths than cancer and cost the global economy over $100 trillion^2^. The potential impact of AMR goes beyond just healthcare, threatening many of the United Nations Sustainable Development Goals. We launched an AMR collection to bring attention to the problem of AMR and suboptimal antimicrobial use in human medicine.

Cross-disciplinary interventions are required to reduce the impact of AMR in human health, such as the United Nations General Assembly’s Global Action Plan for AMR^3^. Nations have begun to operationalise the Global Action Plan through the implementation of National Action Plans (NAPs). At the local level, antimicrobial stewardship aims to reduce the transmission of AMR in humans using organisational approaches to promote and monitor the judicious use of antimicrobials to reduce inappropriate antimicrobial use and ensure infection prevention and control (IPC) is implemented.

Despite the global response, there remains a disconnect between NAPs and local implementation^4^. Rates of inappropriate prescribing in humans remain high and the impact of AMR continues to increase^1^.

A topical issue is the impact of conflict and subsequent mass displacement of people on the transmission of AMR^5^. There are reports of multi-drug resistant *Acinetobacter baumannii* during the Iraq war, high rates of carbapenemase-producing *Enterobacterales* isolated from patients transferred to European hospitals following injuries during the Syrian conflict^6^ and high rates of multi-drug resistant bacteria associated with war-related injuries observed within surgical projects during the recent events in Gaza^7^. The disruption caused by conflict has limited capacity to mitigate AMR through system-wide initiatives and access to effective therapies^7^. It has propagated transmission of AMR through disruption to water and sanitation, overcrowding of healthcare facilities, and lack of IPC resources^7^. To help mitigate the effect of conflict on AMR, there is a need for global collaboration, optimal screening and surveillance systems, and the development of effective IPC practice^5^.

Climate change is another global health crisis impacting the emergence and spread of AMR pathogens. Increasing global temperature has been associated with the emergence and global outbreaks of *Candida auris*, a multi-drug resistant yeast that can survive at higher temperatures compared to other *Candida species*^8^.

Understanding the role of the built environment and IPC in emergence and transmission of AMR is vital. Healthcare is a significant driver, reservoir, and amplifier of AMR^9^. Recent data demonstrate the rapid establishment of AMR pathogens in a newly built intensive care unit environment over a 12-month period^10^. Reservoirs were established within sink drains and dynamic transmission of clonal isolates observed between the environment and humans^10^. One recent study demonstrated that 41% of neonates in a Gambian neonatal intensive care unit acquired an AMR pathogen by day 7 of life with 85% estimated to be new, environmental acquisitions^11^.

The importance of IPC and optimising the built environment to prevent the spread of AMR were clearly highlighted by the COVID-19 pandemic. As healthcare capacity became overwhelmed, patient overcrowding and breakdown in IPC practices were associated with significant outbreaks of AMR pathogens^12^. These outbreaks were addressed through re-focusing on the basic principles of breaking the chain of transmission and minimising inappropriate antimicrobial use through antimicrobial stewardship interventions^12^.

Recent steps have been taken to develop frameworks to help guide the development and implementation of diagnostics to support antimicrobial stewardship activities globally^13^. The development of rapid diagnostics that do not rely on traditional laboratory capacity must ensure that their expected added value from implementation is understood and defined based on the local context in which they will be deployed^13^.

The role of the microbiology laboratory and the interface with clinicians is evolving to use routinely available clinical data to support decision making^13^.The application of artificial intelligence can also support personalised antimicrobial use^14^. As evidence of the potential impact of artificial intelligence decision support tools emerge, we need to ensure that strategies for data collection, regulation, implementation, and oversight keep pace with technology development to ensure that tools provide fair and equitable support to clinicians^15^.

A common theme through all interventions to address AMR is the central role that behavioural sciences play in helping us understand and address actions that potentiate this problem. Understanding the context, culture, and behaviours behind all aspects of decision-making that can contribute to AMR are central to the design and implementation of new approaches to address this issue^4^. A central question remains whether using the terminology AMR is appropriate language for communicating risk compared to different healthcare challenges, such as cancer or Ebola. A recent survey of 1161 people highlighted that the term AMR appeared unsuitable for public communication, with terms such as drug-resistant infection performing better when communicating risk^16^. As evidence continues to emerge surrounding effective methods of communication, it will be vital to ensure that research and policy adapts to reflect this.

With the challenges faced by the global economy and competing healthcare technologies, potential new interventions to support antimicrobial prescribing, IPC, and wider policy approaches need to be evaluated in a way that allows a value-based assessment compared to other areas of healthcare, such as chemotherapy, surgical interventions, and biological agents. Current approaches fail to account for the inclusion of societal and wider economic impact, which are likely to be significantly greater for factors addressing AMR when compared to patient-targeted therapies for conditions such as cancer.

Whilst AMR is a serious threat to global health and security, the global response to address this problem in both human and wider environmental and agricultural sectors provides optimism. Whilst AMR is something that we will face for as long as we rely on antibiotics for the treatment and prevention of infection, research in the areas we showcase in our AMR collection provides optimism that we will be able to respond and mitigate its potential impact on society.

## References

[1] Murray, C. J. L. et al. Global burden of bacterial antimicrobial resistance in 2019: a systematic analysis. *Lancet* **399**, 629–655 (2022). 35065702 10.1016/S0140-6736(21)02724-0 PMC8841637

[2] O’Neill, J. Tackling drug-resistant infections globally: final report and recommendations. Review on antimicrobial resistance. (2016).

[3] United Nations. Draft political declaration of the high-level meeting of the general assembly on antimicrobial resistance. 16108 (2016).

[4] Charani, E. et al. Optimising antimicrobial use in humans—review of current evidence and an interdisciplinary consensus on key priorities for research. *Lancet Reg. Health Eur.* **7**, 100161 (2021). 34557847 10.1016/j.lanepe.2021.100161 PMC8454847

[5] Pallett, S. J. C. et al. The contribution of human conflict to the development of antimicrobial resistance. *Commun. Med.* **3**, 153 (2023). 37880348 10.1038/s43856-023-00386-7 PMC10600243

[6] Abbara, A. et al. Antimicrobial resistance in the context of the Syrian conflict: drivers before and after the onset of conflict and key recommendations. *Int. J. Infect. Dis.* **73**, 1–6 (2018). 29793039 10.1016/j.ijid.2018.05.008

[7] Moussally, K., Abu-Sittah, G., Gomez, F. G., Fayad, A. A. & Farra, A. Antimicrobial resistance in the ongoing Gaza war: a silent threat. *Lancet* **402**, 1972–1973 (2023). 37952545 10.1016/S0140-6736(23)02508-4

[8] Magnano San Lio, R., Favara, G., Maugeri, A., Barchitta, M. & Agodi, A. How antimicrobial resistance is linked to climate change: an overview of two intertwined global challenges. *Int. J. Environ. Res. Public Health* **20**, 1681 (2023). 36767043 10.3390/ijerph20031681 PMC9914631

[9] Cocker, D. et al. Healthcare as a driver, reservoir and amplifier of antimicrobial resistance: opportunities for interventions. *Nat. Rev. Microbiol.*10.1038/s41579-024-01076-4 (2024) 10.1038/s41579-024-01076-4 39048837

[10] Sukhum, K. V. et al. Antibiotic-resistant organisms establish reservoirs in new hospital built environments and are related to patient blood infection isolates. *Commun. Med.* **2**, 62 (2022). 35664456 10.1038/s43856-022-00124-5 PMC9160058

[11] Bah, S. Y. et al. Acquisition and carriage of genetically diverse multi-drug resistant gram-negative bacilli in hospitalised newborns in The Gambia. *Commun. Med.* **3**, 79 (2023). 37270610 10.1038/s43856-023-00309-6 PMC10239441

[12] Rawson, T. M., Ming, D., Ahmad, R., Moore, L. S. P. & Holmes, A. H. Antimicrobial use, drug-resistant infections and COVID-19. *Nat. Rev. Microbiol.* **18**, 409–410 (2020). 10.1038/s41579-020-0395-y PMC7264971 32488173

[13] Moore, L. S. P. et al. Rapid diagnostic test value and implementation in antimicrobial stewardship across low-to-middle and high-income countries: a mixed-methods review. *Infect. Dis. Ther.* **12**, 1445–1463 (2023). 37261612 10.1007/s40121-023-00815-z PMC10233178

[14] Rawson, T. M., Ahmad, R., Toumazou, C., Georgiou, P. & Holmes, A. H. Artificial intelligence can improve decision-making in infection management. *Nat. Hum. Behav.* **1**10.1038/s41562-019-0583-9 (2019). 10.1038/s41562-019-0583-9 31190023

[15] Rawson, T. M. et al. Using digital health technologies to optimise antimicrobial use globally. *Lancet Digit. Health* **6**, e914–e925 (2024). 39547912 10.1016/S2589-7500(24)00198-5

[16] Krockow, E. M., Cheng, K. O., Maltby, J. & McElroy, E. Existing terminology related to antimicrobial resistance fails to evoke risk perceptions and be remembered. *Commun. Med.* **3**, 149 (2023). 37880476 10.1038/s43856-023-00379-6 PMC10600229

